# The relationships between leptin levels in maternal serum and breast milk of mothers and term infants

**DOI:** 10.1080/07853890.2021.1964037

**Published:** 2021-08-11

**Authors:** Feray Çağiran Yilmaz, Ayşe Özfer Özçelik

**Affiliations:** Department of Nutrition and Dietetics, Faculty of Health Sciences, Firat University, Elazig, Turkey

**Keywords:** Breast milk, anthropometry, infant, nutrition status, leptin

## Abstract

**Background:**

Leptin in breast milk play a significant role in metabolic programming.

**Objective:**

The aim of this study to evaluate the relationship between leptin levels in maternal serum and breast milk, and certain anthropometric measurements of infants and mothers.

**Methods:**

This study was conducted with 65 females and term infants. Anthropometric measurements of the mothers and the infants were obtained on the first, third, and sixth months, and leptin levels in maternal serum and breast milk were measured.

**Results:**

In this study, leptin levels in breast milk were positively correlated with leptin levels in maternal serum while the leptin levels in breast milk were lower than the serum leptin levels. It was also determined that mothers’ body weights, BMI values, waist and hip circumferences were increased in a statistically significant way in terms of the months (*p* < .001). It was discovered that all the anthropometric measurements of the mothers were positively correlated with leptin levels in breast milk and maternal serum in all the months (*p* < .001). Furthermore, it was determined that the body weights of the infants in certain months were negatively correlated with leptin levels in breast milk and maternal serum while the lengths of the infants were positively correlated with leptin levels in breast milk and maternal serum (*p* < .05).

**Conclusions:**

This study was determined that leptin levels in breast milk and maternal serum were related to anthropometric measurements of both mothers and infants. Future studies with larger populations are needed to understand the long-term consequences of leptin metabolism comprehensively.

**Key messages**
- This study was determined that breast milk leptin level and maternal serum leptin level demonstrated a positive correlation, and breast milk leptin level was lower than maternal serum leptin level.
- Mothers' anthropometric measurements were positively correlated with leptin levels in breast milk and maternal serum in all the months.
- There was mostly a negative correlation between breast milk leptin level and infant body weight.

## Introduction

Feeding with breast milk for the first six months is the most ideal approach for the development of infants. Bioactive compounds in breast milk, such as leptin, play a significant role in metabolic programming [1–3].

Leptin, which is derived from the Greek word “leptos (thin)” [4], is an adipokine that provides the regulation of body weight and the stimulation of energy expenditure by suppressing the appetite centre [5]. Zhang et al. defined leptin as an adipocyte-derived hormone, which is encoded by the Ob gene, containing 167 amino acids with 16-kDa weight. Leptin, which is fundamentally synthesized by white adipose tissue in proportion to body fat mass, binds to leptin receptors located in the hypothalamus by crossing the blood-brain barrier after being released from adipose tissue into the bloodstream. After binding to the receptors, leptin affects the release of anorexigenic peptides. In this way, leptin reduces food intake and increases energy expenditure. While excess leptin provides energy expenditure by suppressing food intake and increasing thermogenesis, decreases in leptin increase the storage in adipocytes [6]. In a study conducted by Schwartz et al., it was determined for the first time that plasma leptin levels were positively correlated with BMI values and total body fat [7]. Following the discovery that leptin is produced in the placenta and breast epithelium in addition to adipose tissue, studies on breast milk and infant development have gained momentum [8–12].

In addition to meeting the macronutrient and micronutrient needs of newborns, breast milk has a unique value thanks to containing factors such as enzymes, immune components, and adipokine. Adipokines in breast milk, such as leptin, ghrelin, adiponectin, resistin, and obestatin, have been defined in recent years. These adipokines are involved in growth and development in the infancy period and the regulation of energy balance in childhood and adulthood periods. Since its discovery in breast milk, the effects of leptin on neonatal development have been a matter of curiosity.^10^ It is now known that leptin is synthesized by the mammary glands and secreted into breast milk and that leptin passes from the mother's plasma to the milk. In the central nervous system, leptin exhibits anorexigenic effects and has an appetite-reducing effect [13].

Due to all these effects of leptin, in this prospective study, it was aimed to evaluate the relationship between leptin levels in maternal serum and breast milk, and certain anthropometric measurements of infants and mothers.

## Material and method

### Study design and data collection

Preliminary interviews were conducted with the mothers in the last trimester of their pregnancy, and the mothers were informed about the study. A total of 65 volunteer healthy lactating mothers and their breastfeeding infants referred to private maternity hospital were enrolled in this cross-sectional study. The mother who gave birth to term infants (>37th gestational week, >2.5 kg) and who thought to breastfeed their infants for at least six months were called by phone, and those who wanted to participate in the study voluntarily were given appointments. The mothers, together with their infants, were asked to come to the hospital in the morning on empty stomachs (at least 8 h of fasting). The mothers who had a history of gestational diabetes, who were taking medications, who have consumed alcohol or smoked tobacco and the preterm babies and the babies who fed formula were excluded this study. Before initiating the study, the voluntary consent forms were signed by the mothers, and an ethics committee approval was obtained from the Non-Invasive Research Ethics Committee at Fırat University (Approval Date: 06.12.2018; No: 20/11).

### Anthropometric measurements

The anthropometric measurements of the study were collected to evaluate the nutritional status of the subjects. Anthropometric measurements of the mothers and infants were collected by the researchers at the first, third, and sixth months after birth. The weight measurements of the mothers were conducted by using electronic scales with the closest interval of 0.1 kg while their heights were measured with light clothes and without shoes by using a wall-mounted stadiometer with the closest interval of 0.1 cm. The body mass index was calculated according to the WHO standards. The underweight category covered the BMI values less than 18.5 kg/m^2^ while the normal weight category covered the BMI values between 18.5 and 24.9 kg/m^2^. The BMI values that were between 25.0 and 29.9 kg/m^2^ were categorized as overweight while the BMI values that exceeded 29.9 kg/m^2^ were affected by obesity considering the WHO growth reference data [14]. For the waist circumference of mothers, the circumference of the hip is determined between the lowest rib and the iliac crest. Then, the circumference passing through the midpoint is measured while the mother turned to the side and measured for the highest point. The upper-middle arm circumference is measured by accounting for the circumference passing through the midpoint between the acromial and olecranon protrusions on the shoulder while the arm is bent at 90 degrees [15].

Body weight, length, head circumference, and chest circumference of the infants were recorded according to their gestational ages. The length, body weight, head and chest circumference data of the infants at birth were obtained from the hospital records. The body weights of the infants were measured with a standard infant scale, which was sensitive to 0.1 grams, while the infants were completely undressed. The lengths of the infants were measured in the supine position with an infantometer and a special length measuring table with a tape measure on one side and a movable part applied to the infant's feet. Head and chest circumferences were measured with a non-stretch tape measure. The head measurements were conducted while the infant looks straight ahead, the view is perpendicular to the body, the head is in the Frankfurt plane, the tape measure is above the eyebrows (supra-orbital line) in the front, from the uppermost point (occipital protrusion) in the back. On the other hand, the chest circumference measurements were conducted from the breast level by measuring the mid-inspiration. Measurements were recorded in centimetres (cm) and with a sensitivity of 0.1 cm [15]. The WHO Anthro (version 3.2.2) software was used to calculate the percentile for body weight, length, and head circumference of infants by gestational age. The measurements were classified according to percentile intersections. In the study, three weight categories were formed, which covered normal (>15th percentile to <85th percentile), overweight (>85th percentile to <97th percentile), and infants with obesity categories (≥97th percentile) [16].

### Collection, storage, and analysis of the samples

Breast milk samples were collected on three different dates. The first set of samples were collected at 20–30 days after birth (1st month) while the second set of samples were collected within the third month after birth. Finally, the third set of samples were collected in the sixth month after birth and before initiating complementary feeding. At least two hours after the mothers breastfed their infants, 10 mL of breast milk sample was collected from a single breast with an electronic milking machine (Mamajoo Inc., Germany). The breast milk samples were taken to the laboratory where the analyses were performed as quickly as possible in sterile plastic containers that were not exposed to light, and the samples were frozen at −80 °C before the analyses. Samples were thawed one day before the analyses at 4–6 °C. Furthermore, to determine the serum leptin levels of mothers, 3–4 mL of blood samples were drawn into standard biochemistry tubes and centrifuged at 3000 rpm for 5 min at room temperature. Leptin levels in maternal serum and breast milk were measured by using the Human Leptin ELISA Kit (Catalog No: YLA1318HU).

### Statistical analysis of the data

In the analysis of the data, Statistical Package for the Social Sciences-26 (SPSS-26) package software was used. The Kolmogorov Smirnov and Shapiro Wilk tests were conducted to determine whether the quantitative data were normally distributed, and the repeated measures ANOVA test was used to evaluate the means of three repetitive measurements with normal distributions. The median values of three repetitive measurements that did not demonstrate normal distribution and the difference between the 75th and 25th quarters (interquartile range, IQR) were presented, and the Friedman ANOVA test was conducted. Bonferroni and Wilcoxon *post-hoc* analyses were conducted to determine which groups caused the difference between the three measurements. The Spearman Correlation test was conducted to obtain information about the strength and direction of the linear relationship between the two continuous variables that were not distributed normally. In the statistical tests, the confidence interval was regarded as 95%.

## Results

In the study, the descriptive data of the mothers were presented in Table 1. It was determined that the mean age of the mothers was 31.5 ± 5.6 years, and the income level of 53.8% of the mothers was at the moderate level. It was determined that the mean week of gestation of the participants was 38.4 ± 1.1 weeks, and only 19.0% of the mothers were university graduates. When the delivery methods of the mothers were examined, it was determined that 67.7% gave birth by caesarean section while 32.3% of the mothers gave birth by normal birth.

**Table 1.** Descriptive statistics of the mothers.

Age, (mean ± standard deviation)	31.5 ± 5.6	
Income level, *N* (%)	Low	18 (27.7)
	Moderate	35 (53.8)
	High	12 (18.5)
Maternal education, *N* (%)	High school and lower	46 (70.8)
	Graduate and above	19 (29.2)
Week of gestation, (mean ± standard deviation)	38.4 ± 1.1	
Type of delivery, *N* (%)	Normal delivery	21 (32.3)
	Caesarean delivery	44 (67.7)

Certain values of the mothers' anthropometric measurements in the first six months were presented in Table 2. Body weight, BMI values, waist circumferences, hip circumferences of all the women in the study demonstrated a statistically significant increase in terms of the months (*p* < .05). On the other hand, increases were not observed only in the upper-middle arm circumference of the mothers (*p*>.05).

**Table 2.** Anthropometric measurements of the mothers.

Certain values of anthropometric measurements	1st Month X ± SD	3rd Month X ± SD	6th Month X ± SD	p*
Body weight (kg)	75.5 ± 9.7	76.2 ± 10.1	77.1 ± 10.3	<.001
Height (cm)	162.8 ± 5.2	162.8 ± 5.2	162.8 ± 5.2	–
Body mass index (kg/m²)	28.5 ± 4.1	28.8 ± 4.3	29.0 ± 4.5	<.001
Waist circumference (cm)	94.2 ± 12.7	98.1 ± 12.6	99.3 ± 14.9	<.001
Hip circumference (cm)	105.8 ± 9.3	106.3 ± 9.2	107.7 ± 9.3	.018
Upper middle arm circumference (cm)	29.9 ± 2.2	29.2 ± 2.3	29.8 ± 2.4	.056

*Repeated measures ANOVA.

In Table 3, the anthropometric measurements of the infants in the first six months were presented. It was determined that the body weights, lengths, head circumferences, and chest circumferences of the infants were increased in a statistically significant way in terms of the months (*p* < .05).

**Table 3.** Anthropometric measurements of the infants in the first six months.

Certain values of anthropometric measurements	At birth	1st Month Median (IQR)	3rd Month Median (IQR)	6th Month Median (IQR)	p*
Body weight (kg)	3.6 (0.25)	4.8 (0.7)	6.1 (1.2)	7.4 (1.8)	<.001
Length (cm)	50.8 (2.0)	50.9 (5.0)	60.8 (6.5)	66.8 (6.5)	<.001
Head circumference (cm)	34.3 (1.0)	35.1 (2.0)	40.9 (2.0)	44.0 (2.0)	<.001
Chest circumference (cm)	33.1 (2.0)	36.1 (3.3)	42.9 (1.8)	50.0 (2.0)	<.001

*Friedman ANOVA test.

Maternal median serum leptin level was determined to be 1.400 ng/mL in the first month while this value decreased to 1.300 ng/mL in the third month and to 1.027 ng/mL in the sixth month. Accordingly, this decrease was found to be statistically significant (*p* < .001). It was also determined that the level of leptin in breast milk was decreased in a statistically significant way in terms of the months (*p* < .001). Furthermore, it was determined that the leptin level in breast milk was lower than the serum leptin level (Table 4).

**Table 4.** Leptin levels in maternal serum and breast milk.

Leptin level (ng/mL)	1st Month	3rd Month	6th Month	p*
Maternal serum [Median (IQR)]	1.400 (1.069)	1.300 (0.541)	1.027 (0.567)	<.001
Breast milk [Median (IQR)]	0.208 (0.020)	0.148 (0.070)	0.088 (0.064)	<.001

*Friedman ANOVA test.

The relationship between the anthropometric measurements of the mothers and the leptin levels in breast milk and maternal serum were presented in Table 5. Accordingly, it was determined that mothers' body weights, BMI values, waist circumferences, hip circumferences, and upper-middle arm circumferences were positively correlated with leptin levels in breast milk and maternal serum in all the months, and these correlations were statistically significant (*p* < .05).

**Table 5.** Correlations between mother’s anthropometric measurements, and leptin levels in breast milk and maternal serum.

	Leptin level in breast milk						Leptin level in maternal serum					
	1st Month		3rd Month		6th Month		1st Month		3rd Month		6th Month	
	*r*	p*	*r*	p*	*r*	p*	*r*	p*	*r*	p*	*r*	p*
Body weight (kg)												
1st Month	0.687	**.000**	0.735	**.000**	0.737	**.000**	0.533	**.000**	0.695	**.000**	0.826	**.000**
3rd Month	0.694	**.000**	0.741	**.000**	0.744	**.000**	0.580	**.000**	0.702	**.000**	0.800	**.000**
6th Month	0.686	**.000**	0.706	**.000**	0.709	**.000**	0.580	**.000**	0.694	**.000**	0.788	**.000**
BMI (kg/m^2^)												
1st Month	0.758	**.000**	0.832	**.000**	0.832	**.000**	0.564	**.000**	0.767	**.000**	0.918	**.000**
3rd Month	0.773	**.000**	0.842	**.000**	0.842	**.000**	0.576	**.000**	0.783	**.000**	0.888	**.000**
6th Month	0.733	**.000**	0.775	**.000**	0.775	**.000**	0.582	**.000**	0.743	**.000**	0.854	**.000**
Waist circumference (cm)												
1st Month	0.509	**.000**	0.443	**.000**	0.439	**.000**	0.480	**.000**	0.515	**.000**	0.654	**.000**
3rd Month	0.483	**.000**	0.401	**.001**	0.396	**.001**	0.452	**.000**	0.490	**.000**	0.623	**.000**
6th Month	0.501	**.000**	0.399	**.001**	0.396	**.001**	0.421	**.000**	0.505	**.000**	0.65	**.000**
Hip circumference (cm)												
1st Month	0.371	**.002**	0.251	**.044**	0.371	**.002**	0.332	**.007**	0.575	**.000**	0.575	**.000**
3rd Month	0.397	**.001**	0.280	**.024**	0.398	**.001**	0.360	**.003**	0.571	**.000**	0.571	**.000**
6th Month	0.398	**.001**	0.258	**.038**	0.398	**.001**	0.327	**.008**	0.575	**.000**	0.575	**.000**
Upper middle arm circumference (cm)												
1st Month	0.451	**.000**	0.441	**.000**	0.446	**.000**	0.172	.171	0.448	**.000**	0.437	**.000**
3rd Month	0.418	**.001**	0.435	**.000**	0.434	**.000**	0.189	.131	0.419	**.001**	0.335	**.006**
6th Month	0.383	**.002**	0.378	**.002**	0.376	**.002**	0.114	.364	0.385	**.002**	0.339	**.006**

*Spearmen correlation test.

In Table 6, the relationship between anthropometric measurements of infants, and the leptin levels in breast milk and maternal serum were demonstrated. It was determined that the body weights of the infants in the third month and the breast milk leptin level in the third and sixth months were negatively correlated while the body weight of infants in the sixth month and the breast milk leptin level in the first, third and sixth months were also negatively correlated. Additionally, it was determined that the body weights of infants in the sixth month were negatively correlated with maternal serum leptin levels in the first and third months. Accordingly, all of these correlations were statistically significant. It was also determined that there was a positive correlation between the length of the infants and leptin levels in breast milk and maternal serum, which was statistically significant. Finally, there was no statistically significant difference between infants’ head circumferences and chest circumferences, and the leptin levels in breast milk and maternal serum (*p*>.05).

**Table 6.** Correlations between anthropometric measurements of infants and leptin levels in breast milk and maternal serum.

	Leptin level in breast milk						Leptin level in maternal serum					
	1st Month		3rd Month		6th Month		1st Month		3rd Month		6th Month	
	*r*	p*	*r*	p*	*r*	p*	*r*	p*	*r*	p*	*r*	p*
Body weight (kg)												
1st Month	0.004	.974	−0.059	.639	−0.059	.643	−0.141	.262	0.005	.969	0.236	.058
3rd Month	−0.168	.181	−0.262	**.035**	−0.261	**.036**	−0.220	.078	−0.165	.189	0.107	.398
6th Month	−0.304	**.014**	−0.525	**.000**	−0.526	**.000**	−0.307	**.013**	−0.305	**.013**	−0.078	.539
Length (cm)												
1st Month	0.530	**.000**	0.420	**.000**	0.424	**.000**	0.418	**.001**	0.535	**.000**	0.669	**.000**
3rd Month	0.522	**.000**	0.379	**.002**	0.383	**.002**	0.404	**.001**	0.528	**.000**	0.674	**.000**
6th Month	0.492	**.000**	0.405	**.001**	0.408	**.001**	0.403	**.001**	0.500	**.000**	0.689	**.000**
Head circumference (cm)												
1st Month	−0.100	.427	−0.203	.105	−0.205	.101	−0.149	.236	−0.099	.432	−0.108	.392
3rd Month	−0.112	.375	−0.223	.074	−0.221	.076	−0.128	.311	−0.111	.380	−0.118	.349
6th Month	−0.073	.563	−0.206	.100	−0.208	.097	−0.224	.073	−0.073	.562	−0.086	.498
Chest circumference (cm)												
1st Month	0.078	.535	−0.085	.500	−0.081	.522	−0.004	.976	0.082	.519	−0.115	.364
3rd Month	−0.112	.375	−0.223	.074	−0.221	.076	−0.082	.518	−0.111	.380	−0.118	.349
6th Month	−0.073	.563	−0.206	.100	−0.208	.097	−0.095	.451	−0.073	.562	−0.086	.498

*Spearman correlation test.

## Discussion

Breast milk has a unique value because it meets the micronutrient and macronutrient needs of newborns thanks to containing factors such as enzymes, immune components, and adipokine. The leptin found in breast milk suppresses appetite and regulates calorie intake. Since the discovery of leptin in breast milk, the effects of leptin on neonatal development have been a matter of curiosity [10,17,18]. In this study, it was aimed to evaluate the relationship between the leptin levels in maternal serum and breast milk, and the anthropometric measurements of mothers and infants. This study is the important study that covers a high number of samples, with 6-month follow-ups, in which anthropometric measurements of mothers and infants, in addition to their body weights, are evaluated at the same time.

In several previous studies, it was determined that breast milk leptin level and maternal serum leptin level demonstrated a positive correlation, and breast milk leptin level was lower than maternal serum leptin level [19–21]. Ucar et al. reported that breast milk leptin level was positively correlated with maternal serum leptin level, and breast milk leptin level was determined to be lower than maternal serum leptin level [22]. In another study, Casabiell et al. reported that breast milk leptin level was positively correlated with maternal serum leptin level, and breast milk leptin level was determined to be lower than maternal serum leptin level [23]. Similar to other results, in the current study, it was determined that breast milk leptin level demonstrated a positive correlation with maternal serum leptin level, and breast milk leptin level was lower than maternal serum leptin level.

Leptin, which is known to be released mostly from adipose tissue, was found to be associated with the BMI value of mothers in previous studies [23,24]. Several studies discovered a negative relationship between breast milk leptin level and infant development [17,25–27]. Miralles et al. followed 28 non-obese mothers and their infants, who were fed with breast milk for at least 6 months. In their study, the researchers determined that breast milk leptin level was positively correlated with maternal serum leptin level and maternal BMI while being negatively correlated with infant development [16]. Schuster et al. followed 23 mothers and their infants for 6 months, and it was reported that there was a positive relationship between the BMI value of the mothers and leptin levels in breast milk and maternal serum while there was a negative relationship between breast milk leptin level and the weight gained by the infants at the end of the 6th month [27]. Logan et al., discovered a positive correlation between breast milk leptin level and maternal BMI [28]. In a study conducted by Savino et al., 58 mothers and their infants were followed. Accordingly, the researchers reported that maternal serum leptin had a positive relationship with the BMI value of the mothers [19]. Chan et al. examined the leptin content of breast milk collected in the 4th month of lactation and the anthropometric measurements of the infants. In the study, the BMI value of mothers and breast milk leptin levels were found to be positively correlated, while it was determined that the body weight of infants according to length was negatively correlated with breast milk leptin level [25]. In a three-month follow-up study with 50 mothers and newborns, Uysal et al., determined that breast milk leptin level was positively correlated with the BMI value of the mother, but not with the newborn BMI value [29]. Bielicki et al., conducted a 6-week follow-up study with 33 mothers, 24 term infants, and 9 preterm infants, and discovered that there was a positive relationship between maternal serum leptin level and maternal BMI while there was no correlation between breast milk leptin level and newborn weight and maternal BMI value [30].

Similar to numerous studies in the literature, in the current study, it was determined that mothers' body weight, BMI value, waist circumference, hip circumference, and upper-middle arm circumference were positively correlated with leptin levels in breast milk and maternal serum in all the months. Accordingly, this relationship was statistically significant. When leptin levels in breast milk and maternal serum, and the development of infants were examined, it was determined the body weight of infants in the 3rd month and the breast milk leptin level in the 3rd and 6th months were negatively correlated while the body weight of infants in the 6th month and the breast milk leptin level in the 1st, 3rd and 6th months were also negatively correlated. Furthermore, the maternal serum leptin level in the 1st and 3rd months were negatively correlated with the body weight of infants in the 6th month. Accordingly, all these correlations were statistically significant. It was determined that there is a positive correlation between the length of the infants and leptin levels in breast milk and maternal serum, which was statistically significant. There was no statistically significant difference between infants' head circumference and chest circumference, and leptin levels in breast milk and maternal serum.

### Limitations

In this study, the relationship between leptin levels in maternal serum and breast milk and certain anthropometric measurements of infants and mothers were evaluated. This study has several limitations. We did not measure serum leptin at the same age time in all subjects enrolled. Further, we were unable to measure fat mass at the same time of leptin sampling in mothers and infants. In future studies, it can be beneficial to conduct investigations in which other adipokines in breast milk are examined, infants are followed up at later ages, the number of samples is higher and preterm and multiple pregnancies are included.

## Results

This study supports the fact that breastfeeding in the first six months of life is critical to the development of the infant. This study provides some evidences for potential association of maternal serum and milk levels of leptin with infant’s body weight. Anthropometric measurements of mothers and infants are associated with breast milk leptin levels and maternal serum leptin levels, and longer follow-up studies with larger samples are necessary to understand this issue comprehensively.

## Data Availability

The present study was approved by the Ethics Committee of Fırat University. Datasets used or analyzed during the current study are available from the corresponding author on reasonable request.
